# CRISPR-Cas9 Mediated Gene-Silencing of the Mutant Huntingtin Gene in an In Vitro Model of Huntington’s Disease

**DOI:** 10.3390/ijms18040754

**Published:** 2017-04-02

**Authors:** Nivya Kolli, Ming Lu, Panchanan Maiti, Julien Rossignol, Gary L. Dunbar

**Affiliations:** 1 Field Neurosciences Institute laboratory for Restorative Neurology at Central Michigan University, Mt. Pleasant, MI 48859, USA; kolli1n@cmich.edu (N.K.); lu1m@cmich.edu (M.L.); maiti1p@cmich.edu (P.M.); rossi1j@cmich.edu (J.R.); 2 Program in Neuroscience, Central Michigan University, Mt. Pleasant, MI 48859, USA; 3 Department of Psychology, Central Michigan University, Mt. Pleasant, MI 48859, USA; 4 Field Neurosciences Institute, St. Mary’s of Michigan, Saginaw, MI 48604, USA; 5 Department of Biology, Saginaw Valley State University, Saginaw, MI 48604, USA; 6 College of Medicine, Central Michigan University, Mt. Pleasant, MI 48859, USA

**Keywords:** Huntington’s disease, CAG repeat, mutant huntingtin, gene editing, CRISPR-Cas9 system, pattern of NHEJ, YAC128, Kozak sequence

## Abstract

Huntington’s disease (HD) is a fatal neurodegenerative genetic disease characterized by a loss of neurons in the striatum. It is caused by a mutation in the Huntingtin gene (*HTT*) that codes for the protein huntingtin (HTT). The mutant Huntingtin gene (m*HTT*) contains extra poly-glutamine (CAG) repeats from which the translated mutant huntingtin proteins (mHTT) undergo inappropriate post-translational modifications, conferring a toxic gain of function, in addition to its non-functional property. In order to curb the production of the mHTT, we have constructed two CRISPR (clustered regularly interspaced short palindromic repeat)-Cas9 (CRISPR associate protein) plasmids, among which one nicks the DNA at untranslated region upstream to the open reading frame (uORF), and the other nicks the DNA at exon1-intron boundary. The primary goal of this study was to apply this plasmid into mesenchymal stem cells (MSCs) extracted from the bone-marrow of YAC128 mice, which carries the transgene for HD. Our results suggest that the disruption of uORF through CRISPR-Cas9 influences the translation of mHTT negatively and, to a lesser extent, disrupts the exon1-intron boundary, which affects the translation of the mHTT. These findings also revealed the pattern of the nucleotide addition or deletion at the site of the DNA-nick in this model.

## 1. Introduction

Huntington’s disease (HD) is a rare autosomal dominant neurodegenerative genetic disorder, resulting in abnormal movements of the limbs, along with cognitive deficits and psychiatric symptoms [1]. HD is progressive and is characterized by chorea, as well as emotional disturbances, including depression and sleep abnormalities. As the disease progresses, patients have trouble controlling movement of the extremities, which gradually leads to the total loss of the ability to perform day-to-day activities [2]. The life expectancy of the patient with HD is approximately 10 years following the onset of symptoms [1].

The underlying cause of the disease is a genetic mutation of the huntingtin gene (m*HTT*), which codes for the huntingtin protein (HTT), whereby the poly-glutamine (CAG) repeat domain is extended to more than 35 CAG repeats. This leads to the inability of the mHTT to undergo appropriate post-translational changes, thereby rendering it useless in the cell body [3]. Although the hereditary cause for the ailment is known, the exact pathology behind the disease is still under investigation [4]. Many hypotheses have been proposed to explain the molecular mechanisms underlying HD pathogenesis, including mitochondrial dysfunction, loss of brain derived neurotrophic factor (BDNF), as well as excitotoxicity. However, a unifying model to establish the real mechanism has yet to be uncovered. At present, only palliative treatments are available to alleviate the symptomology of the disease, as a cure or an effective treatment has been elusive [5].

Many studies have shown that embryonic stem cells (ESCs) can delay the progression of HD, putatively by providing a protective environment in the striatum, the region in which neurons are first lost as part of the disease pathology [6]. More recently, transplantation of induced pluripotent stem cells (iPSCs), generated from patient’s skin cells, has been proposed as an alternative cell-based therapy for HD [7]. Although allogeneic stem cell transplantation is relatively safe, autologous transplantations provide significantly less immunogenic responses than allogeneic stem cell transplantations [8]. However, since HD is an autosomal dominant neurological disorder, the stem cells generated from the person affected with the HD would carry the heterozygous alleles having the normal *HTT* gene and the m*HTT*. Since the mHTT protein formed from the m*HTT* gene has a toxic gain of the function property, editing the m*HTT* gene in the stem cells, by either silencing the m*HTT* gene or correcting the m*HTT* before using them for autologous transplantation, should prove to be more beneficial than utilizing direct autologous transplantation [9]. This idea has led to the search of gene-editing tools, such as transcription activator-like effector nucleases (TALENs), zinc finger nucleases (ZFNs) and the emerging CRISPR-Cas9, all of which are able to induce a double-strand break in the DNA. Although some recent advancement occurred in the genetic engineering with the advent of ZFNs, meganucleases, and TALENs [10,11], the use of the CRISPR-Cas9 system has the advantage of having a relatively high efficacy and a more cost-effective means of editing the *mHTT* gene [12].

The CRISPR-Cas9 system is an adaptive immune mechanism present in 40% of the sequenced bacterial genomes and 90% of the archaea [13], and has been identified to protect these microbes from the future invasions by bacteriophages [14]. After many years, the essential components of this system, including the guide RNA (gRNA) which is essential to direct the Cas9 protein (an important enzyme for induction of DNA double-strand break) to the targeted region on the genome, have been identified. The CRISPR-Cas 9 system is now broadly applied to eukaryotes for editing genes, including creating knock-in, knock-out, and also to correct the mutated genes in the genome [15]. After the CRISPR-Cas9 system induces a double strand break (DSB) at the targeted site, the endogenous cellular repair mechanisms will be activated, and can naturally attempt to repair and rejoin the broken DNA strands through either of two mechanisms: (i) non-homologous end joining (NHEJ) or (ii) homology-directed repair (HDR) [10,16]. Through NHEJ, insertions and deletions (called indel mutations) of a small number of the nucleotides is possible, and this might trigger a frame-shift mutation which can lead to the “loss-of-function” of protein-coding genes via the disruption of open reading frame (ORF) [10,17,18]. NHEJ-induced mutations can lead to silencing of a gene [19]—whereas, using HDR, a large portion of the gene (2–10 kb) can be deleted and, simultaneously, an incorporation of the exogenous DNA at the target region of the genome is possible [20].

**Figure 1:**
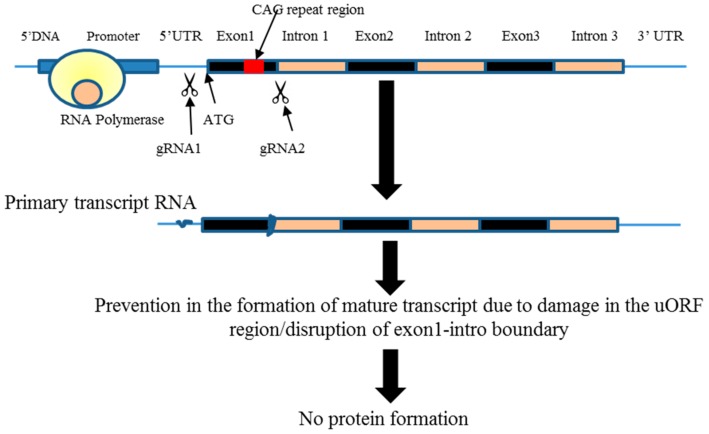
Schematic diagram of the targeted region to induce a double-strand break through CRISPR-Cas9 system on the mutant huntingtin gene (m*HTT*). The target region in the mutant human huntingtin gene is near the start codon. The target region in the mutant human Huntingtin gene is at the junction of the exon1 and intron. Scissors indicate the location of the double-strand break and black color highlights the exon regions in the *HTT* gene and orange color indicate introns.

It was previously reported that 5′ untranslated region (UTR) of the huntingtin gene plays a critical role in regulating the synthesis of the HTT [21]. Particularly, the presence of upstream open reading frame (uORF) within 5′ UTR can affect the translation of the downstream ORF [22,23,24]. Based on this fact, we have hypothesized that the designed CRISPR-Cas9 plasmid could disrupt the uORF present within the 5′ UTR of the mRNA, thus reducing the translation of the mutant huntingtin gene product in the MSCs derived from YAC128 mouse model. It is known that the disruption of the uORF of the gene would activate stop codons as a protective mechanism in order to terminate the transcription of a mutant gene (Figure 1). Given this, we have designed another CRISPR-Cas9 plasmid with the gRNA targeting the exon1-intron boundary and observed the effect of its disruption on the translation of the HTT protein.

The overall goal of this study was to examine the pattern of NHEJ at the 5′ UTR versus exon1-intron boundary, and also to check the efficiency of these two targets in terms of production of the mutant huntingtin.

## 2. Results and Discussion

In this study, it was demonstrated that Lenti-CRISPR-Cas9-mediated silencing of mHTT dramatically reduced the production of mHTT in the bone-marrow-derived (BM) MSCs that were extracted from YAC128 mice, which carry the human mutant HTT transgene. The gene-editing at the untranslated region, and exon1-intron junction, played a role in the transcription and translation of gene. Even though we have found a significant reduction in the gene translation with both the targets, we have observed that targeting untranslated regions of the gene can aid in increased efficiency.

In this study, both the designed Lenti-CRISPRv2-guideRNA1 and Lenti-CRISPRv2-guideRNA2 were non-allele-specific to the human huntingtin gene. Because these gRNAs are specific to human huntingtin gene and cannot target mouse huntingtin gene, we chose the YAC128 transgenic mouse model to explore the extent to which CRISPR-mediated gene-silencing can be achieved when targeting a single allele.

Gene silencing can be achieved in different ways, including: (1) RNA interference (RNAi), which is a form of gene silencing method at the posttranscriptional stage that prevents the translation of protein from mRNA; (2) gene-editing, using transcription activator-like effector nucleases (TALEN), Zinc figure nucleases (ZFN), or CRISPR-Cas9, in which the gene silencing is achieved by blocking the transcription of the gene into mRNA by changing the nucleotide in the gene. When using CRISPR-Cas9, an important factor that needs to be considered is the safety of the gRNA. In silico assessment, using the CRISPR MIT tool [25] for gRNA1, revealed that it is a high-quality target with a score of 60, and the total in silico off-target hits for gRNA1 were 234, among which 44 were in genes. More than 98% of the mismatches have three mismatches (Table S1). For gRNA2, the total number of off-target hits were only 29, among which 13 were in genes. One hundred percent of the mismatches had four mismatches (Table S2). Monteys and colleagues [26] also observed 416 off-target hits with their gRNA, among which 90% of the off-targets have three mismatches. When the top 11 off-targets, having 1 or 2 or 3 mismatches, were mapped in the whole genome, using Sanger sequencing, it was observed that none of the locations had indel mutations caused by CRISPR-Cas9 activity.

### 2.1. Mutant Huntingtin Gene (mHTT) Editing

**Figure 2:**
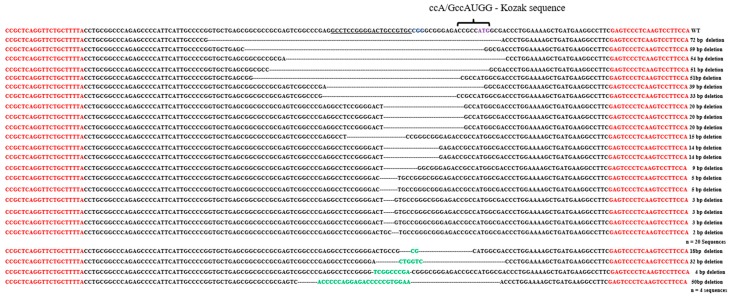
CRISPR-Cas9-mediated editing of *HTT* transgene at the upstream open reading frame (uORF) region. This figure shows the pattern of deletion around the double-strand break (DSB) induced by Lenti-CRISPR-gRNA1. Both addition and deletion of the nucleotides were summarized in this figure, with the Kozak sequence being highlighted. The term “n” refers to the number of the sequences that were analyzed to study the addition and deletion of the nucleotides. The top line shows the amplified product which is 170 bp in length. Red color shows the sequence of forward and reverse primers. Underlined sequence is the gRNA1 location in the *HTT* transgene. The green colored nucleotides are those that were added after induction of a DSB at the targeted site.

**Figure 3:**
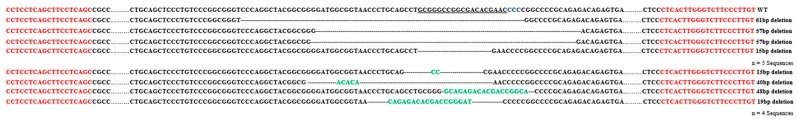
CRISPR-Cas9-mediated editing of *HTT* transgene at the exon1-intron region. This figure shows the pattern of deletions around the DSB induced by Lenti-CRISPR-gRNA2. Both addition and deletion of the nucleotides are summarized. The term “n” refers to the number of the sequences that were analyzed to study the addition and deletion of the nucleotides. The top line shows the amplified product, which is 339 bp in length. The red color shows the sequence of forward and reverse primers. The underlined sequence is the gRNA location in the *HTT* transgene. The green colored nucleotides are the ones which were added nucleotides after induction of a DSB at the targeted site.

The results from the sequencing study have revealed that there was as low as three nucleotide deletions, compared to as many as 72 nucleotide deletions around the gRNA1 sequence (Figure 2) among 24 colonies. Four of the colonies showed both addition and deletion of the nucleotides; gRNA1 was located -14 nucleotides to the Kozak sequence on 5′ (5-prime) of *HTT* gene. It was previously reported that the Kozak sequence plays an important role in the initiation of the translation process [27], and any mutation upstream to the start codon would have a negative effect on the translation of the protein [28]. Next, we have investigated if the CRISPR-Cas9 editing elsewhere, other than the uORF, would result in the large number of nucleotide deletions observed with the gRNA1. For this, we have selected the gRNA2 that targets exon1-intro boundary as shown in (Figure 1). It has to be noted that NHEJ at the exon1-intro might lead to the production of truncated or mis-spliced proteins, which needs to be determined if using this sequence for therapeutic purpose. Upon sequencing, we have observed as high as 61 nucleotide deletions, and as low as 1 nucleotide deletion, when using gRNA2 (Figure 3). These data reveal that both the gRNAs, regardless of the targeted site, resulted in a large number of deletions, and also addition of the nucleotides. Although previous studies have reported short deletions and additions, extensive observations were not made on the pattern of the nucleotide deletion. This is the first study of its kind to investigate and describe comprehensively the pattern of NHEJ post-CRISPR application. These data not only help in performing an efficient gene-silencing, but also facilitate in the design of the HDR strategy to perform knock-in experiments.

### 2.2. Percent Nucleotide Deletion

**Figure 4:**
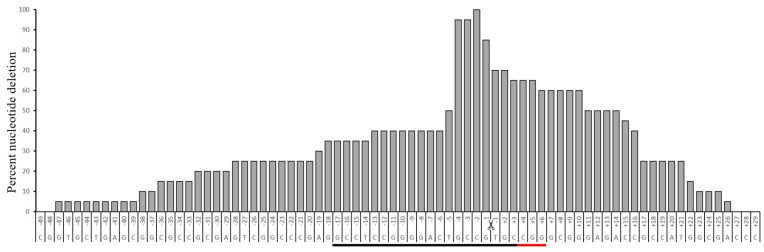
Percent nucleotide deletion after application of CRISPR-Cas9-gRNA1 at the uORF site. This graph highlights the pattern of nucleotide deletion on either side of the double-strand break (DSB) point. The deletion of the neucleotides were observed on both sides of the DSB region. Towards the upstream of DSB, nucleotides are labeled as +1, +2, and so on. Nucleotides towards the downstream of the DSB are labeled as −1, −2, and so on. Underlined in black is the gRNA1 sequence, and in red is the PAM sequence. As can be seen, the greatest percentage of nucleotide deletions are those closest to the DSB-point.

PAM sequence is located downstream to the gRNA sequence. CRISPR-Cas9 induces a DSB at three nucleotides upstream to the PAM location (Figure 4). To study the pattern of nucleotide deletion at the targeted region, twenty colony sequences were analyzed independently, after the application of CRISPR-Cas9-gRNA1. It was observed that the 2nd nucleotide located downstream to the point-of-DSB was deletion in all the colonies. There was deletion of −1 to −3 nucleotides in 95% of the colonies, and +1 to +3 nucleotide deletions in 70% of the colonies. The percent deletion of the nucleotides are skewed towards the upstream of DSB, suggesting that there are more chances that the nucleotides located upstream of the DSB might occur, when compared to the nucleotides located on the downstream of the DSB. In colonies that showed large deletion, it was interesting to observe that the nucleotides located downstream to the DSB were eliminated at higher frequency, when compared to the nucleotides located upstream to the DSB. This information is important and needs to be considered when designing the donor sequence in HDR types of CRISPR-Cas9-mediated editing.

### 2.3. Effect of Gene-Editing on Gene Transcription

**Figure 5:**
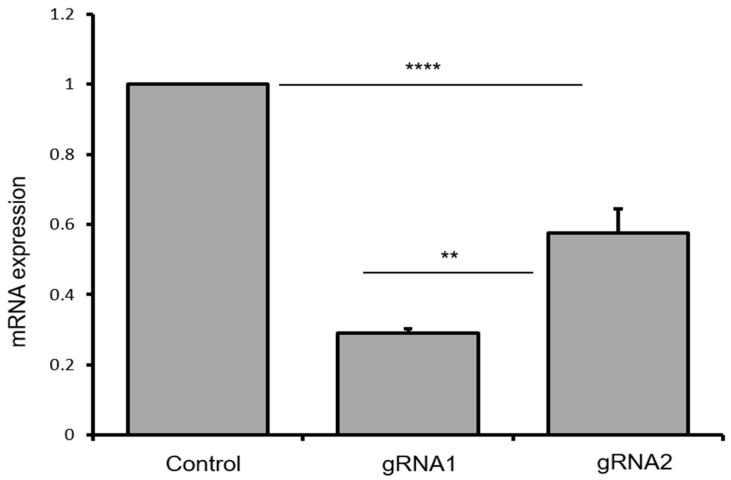
Levels of mRNA from the mutant huntingtin after CRISPR-Cas9 application in BM-MSCs derived from YAC128 mouse. In the puromycin-selected-YAC128 cells, there was a significant reduction (*p* < 0.0000) of m*HTT* (mRNA) expression in gRNA1- and gRNA2-treated cells, relative to controls. There was significantly less mRNA (*p* < 0.002) in gRNA1-treated cells in comparison to gRNA2-treated cells. Results are expressed as mean fold changes in mRNA expression ± SEM. ** *p* < 0.01 and **** *p* < 0.0001.

Quantitative reverse transcriptase-PCR (qRT-PCR) was used to examine the level of mRNA expression in the puromycin-selected cell cultures. Statistical analysis was performed to compare differences in gene expression between control MSCs (carrying transgene) and each of the individual treatment groups (i.e., Lenti-CRISPRv2-gRNA1 and Lenti-CRISPRv2-gRNA2), as well as differences between the three treatment groups (Figure 5). Statistical analyses revealed that targeting both the untranslated region and exon1-intron region of the gene resulted in overall significant difference (F(2, 6) = 227.88, *p* < 0.000). Levels of mRNA were compared between the control MSCs and the groups receiving CRISPR-gRNA1-mediated editing, showing a significant decrease in the expression of mHTT (t(4) = 93.89, *p* < 0.0000). Similarly, there was a significant decrease in mRNA levels between control and gRNA2 (t(4) = 10.54, *p* < 0.000). Interestingly, a significant difference between expression of mRNA levels from m*HTT* gene in CRISPR-gRNA1 and CRISPR-gRNA2 treatment (t(4) = 2.78, *p* < 0.002), was also observed, suggesting that targeting untranslated regions in the gene would be a better choice when compared to targeting exon1-intron junctions to achieve gene silencing.

**Figure 6:**
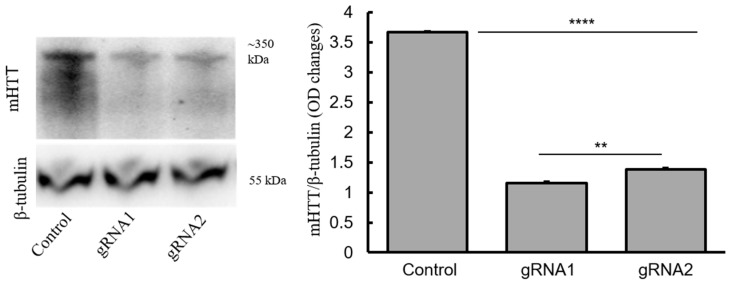
mHTT levels after treatment of CRISP-Cas9 in BM-MSCs. Reduction in the mHTT was observed in the CRISP-Cas9-treated BM-MSCs, compared to untreated cells. The optical density of the mHTT/β-tubulin is shown in the graph. Results are expressed as mean ± SEM from three independent experiments. ** *p* < 0.01 compared to treated groups. There was significantly lower mHTT level in gRNA1-treated cells, in comparison to gRNA2-treated cells. ** *p* < 0.01 and **** *p* < 0.0001.

It was observed that CRISPR-gRNA1 and CRISPR-gRNA2 resulted in ≈79% and ≈58% reduction in mHTT production. Although every cell showed CRISPR-activity, as the NHEJ mechanism of the repair is random, CRISPR-mediated editing did not result in 100% blockade. With gRNA1, it was observed that there was a 21% expression coming from the genes that had fewer nucleotide deletions, or which did not have Kozak sequence disturbed (Figure 6). If this is the case, it may be possible to spare wild-type gene expression to some extent, but also achieve a sufficiently effective blockade in transcription of the mutant allele at the same time. Clearly, a method which can avoid, or at least reduce, the silencing of the normal huntingtin allele, while having a high efficiency in silencing the mutant allele, would be optimal.

### 2.4. Effect of Gene-Editing on Translation

Reduction of HTT in treated MSCs was observed in Western blot (Figure 6), supporting the finding observed in qRT-PCR analysis. Statistical analyses revealed that targeting both the untranslated region and exon1-intron region of the gene resulted in an overall significant difference (F(2, 6) = 9450, *p* < 0.000). Post hoc analysis using Tukey HSD test indicated that there was a significance difference between the gRNA1 and gRNA2 treatments (*p* < 0.05), supporting the finding from the qRT-PCR data.

The actual function of the HTT still remains unclear. Many studies have been conducted to study the impact of silencing both normal and mutant huntingtin alleles, in in vitro and in vivo. It was shown in YAC128 mice that, even a 40% reduction in mutant and wild-type HTT expression potentiated a therapeutic benefit by significant improvement in behavioral deficits [29]. Drouet and colleagues [30] have silenced both mutant and wild-type transcripts efficiently in vivo, and observed that partial inactivation (25%–35%) of normal HTT does not aggravate HD pathology. Other researches have also documented similar results, demonstrating that silencing the wild-type allele (50%) would not influence the survival of neurons in mice [31,32] or humans [33,34]. In addition, there are reasonable arguments that suggest that accentuating long-term unavailability of the HTT in the adult brain would produce a loss-of-function effect, one of which could affect the survivability of the neurons [35,36].

In addition, studies have shown allele-specific silencing of mHTT with RNAi, siRNA, and shRNA, can spare the normal *HTT* gene at an efficiency ranging from a 20%–50% block in translation of the mRNA into the mHTT [37,38]. Unless the mutation-sequence is unique or present only in the abnormal allele, it is not possible to use CRISPR-Cas9 to edit the diseased-allele alone, while sparing the normal allele. In HD and other dominant trinucleotide repeat disorders, such as spinocerebellar ataxia types 1 and 3, as the CAG nucleotide are present in both normal and mutant allele, allele-specificity can only be achieved through the use of existing single nucleotide polymorphisms (SNPs) in the mutant allele. However, the chance that these SNPs exiting in the selected gRNA is much less, making it more challenging to achieve allele specificity.

Most recently, Shin and colleagues [39] used the SNP-based approach to inactivate the mHTT by excising 44 kb deletion from promoter to exon1 region, as a proof of principle. A similar study conducted by Monteys and colleagues [26] also used SNP-based CRISPR-Cas9 targeting in the mHTT, and demonstrated that small deletions induced by NHEJ can, itself, lead to gene silencing of up to 40% in vivo. A novel gene-correction strategy has utilized the CRISPR-Cas9 and *piggyBac* transposon-based approach in human induced pluripotent stem cells (hiPSCs), and demonstrated a reversal of the HD phenotype in the corrected cultured cells [40]. Though our study has also aimed at silencing the mHTT, there are notable differences with previous attempts. First, we have targeted uORF and demonstrated that target-location could play a critical role in achieving greater efficiency when using NHEJ approach. We have extensively studied NHEJ pattern at the DSB, which provides useful information for designing the donor sequence in the HDR approach. Secondly, we have also demonstrated that NHEJ could produce large numbers of nucleotide deletions, which could, itself, lead to gene silencing.

Despite the challenges of designing allele-specific targeting with CRISPR-Cas9, as well as the lack of efficient delivery mechanisms, it is possible to achieve a permanent blockade of the gene transcription within a single application of this type of treatment, unlike the RNAi strategies. The RNAi approach has problems with the long-term expression of the applied RNAi, and because of this reason, these strategies demand frequent injections of the RNAi into the CNS, complicating the clinical utility of this approach for managing chronic diseases [41]. We emphasize that the results from our study indicate that CRISPR-mediated editing at uORF results in a greater gene-silencing, when compared to targeting other regions on the gene.

## 3. Materials and Methods

### 3.1. Experimental Design

To study the CRISPR-Cas9-mediated m*HTT* editing, two CRISPR-Cas9 constructs have been designed. One of these is able to cut the 5′ DNA at the uORF region (Figure 1), which is essential for the formation of the mature RNA. The other construct was designed to target the junction of the exon1-intron region of the m*HTT* (Figure 1). The application of either of these two constructs in MSCs extracted from the bone marrow of YAC128 mouse model of HD, that carries the transgene of mutant human *HTT*, should lead to the disruption of the open reading frame of the m*HTT* gene, further blocking the transcription.

**Figure 7:**
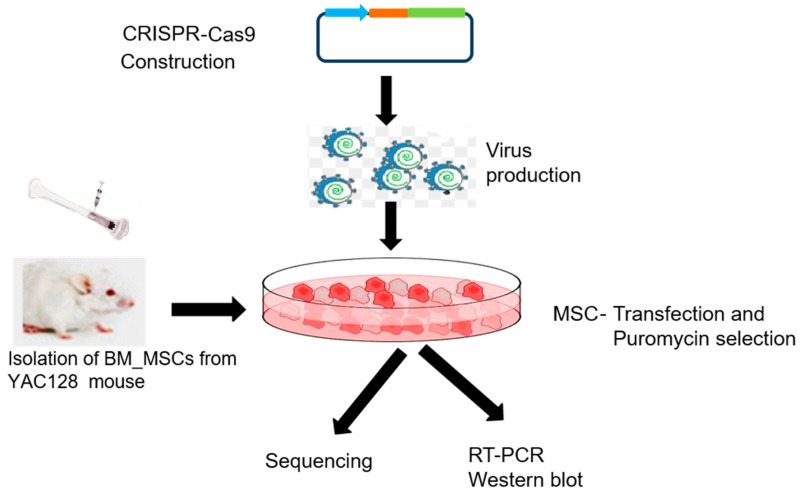
Flowchart representing the methodology for CRISPR-Cas9 mediated silencing of the m*HTT* gene in vitro. Briefly, the constructed CRISPR-Cas9 gRNA was delivered into the cells using lentivirus. CRISPR-Cas9-meadiated gene editing was then confirmed using Sanger di-deoxy nucleotide sequencing method. Using real time polymerase chain reaction (RT-PCR), the transcription of the m*HTT* was analyzed, which was further supported by Western blot to check the level of the mHTT post CRISPR-Cas9 application in the MSCs.

Constructed plasmids are traditionally delivered into the cells via synthetic (e.g., lipofection, polymers, or nanoparticle cargos) or non-synthetic (e.g., virus) methods [42]. In our study, we have used lentivirus. The flow of the experiment involved in this study is diagrammed in Figure 7.

### 3.2. Gene Cloning

In order to clone the target sequence (i.e., gRNA) into the Lenti-CRISPRv2 (Addgene plasmid # 52961, Addgene; Cambridge, MA, USA), two oligos (Table 1) were synthesized to produce a plasmid with the same overhangs following BsmBI digestion. The gRNA was then cloned into the digested Lenti-CRISPRv2 plasmid, using T4 DNA ligase, which was transformed into One Shot^®^ Stbl3™ (Thermo Fischer Scientific; Waltham, MA, USA) chemically competent *Escherichia coli* for the amplification of the plasmid. Ampicillin-resistant colonies were purified using a miniprep plasmid purification kit (Qiagen; Valencia, CA, USA). The DNA was extracted from randomly-picked bacterial colonies and then preliminary screening was performed for identifying successful cloning, using the primers forward 5′-AGATCTTCAACCTCTGGATTACAAAATTTGTG-3′ and reverse 5′-CCTGCAGGGCCCAAAGGGAGATCCGACTC-3′, which amplifies the woodchuck hepatitis virus (WHP) post-transcriptional regulatory element (WPRE) region with a product size of 590 kb. We used the following 3 steps: 95 °C for 5 min for 1 cycle (step 1); 35 cycles of 94 °C for 30 s, 50 °C for 30 s and 72 °C for 45 s (step 2); and 72 °C for 2 min (step 3). The plasmids were further confirmed through restriction-endonuclease-digestion assay. The DNA sequencing service at the Research Technology Support Facility at Michigan State University, USA, was used to detect the presence of gRNA.

### 3.3. Viral Production

For viral production, the HEK293FT cell line (Thermo Fischer Scientific) was plated in Dulbeco modified Eagle’s medium (DMEM; Gibco^®^; Waltham, MA, USA), with 10% deactivated fetal bovine serum (FBS; Gibco^®^; Waltham, MA, USA) and 1% antibiotics (penicillin and streptomycin, Sigma; St. Louis, MO, USA) at 1.5 × 10^6^ cells in 6-well plate (MidSci; St. Louis, MO, USA) before the day of transfection. The following day, the media was removed from the dish and replaced with fresh DMEM with 10% deactivated FBS, without antibiotic. The cloned Lenti CRISPR v2-gRNA1 or Lenti CRISPR v2-gRNA2 was transfected into the HEK293FT cells, along with pCMV-VSV-G (a gift from Bob Weinberg: Add gene plasmid # 8454, Addgene), and the psPAX2 plasmid, which encodes HIV Gag-Pol (psPAX2 was a gift from Didier Trono: Addgene plasmid # 12260, Addgene) at a ratio of 2:1:5, using Lipofectamine^®^ 3000 reagent (Thermo Fischer Scientific), according to the manufacturer’s protocol.

A successful transfection was determined based on the hallmark morphological changes seen after the expression of the VSVG glycoprotein, causing the 293FT cells to fuse, subsequently making them look large like ‘balloon-shaped’, multinucleated cells [43]. Running eGFP plasmids in parallel during transfection helped avoid potential false positives conclusions that can occur when interpretation is based upon the morphological changes (Figure S1). Media was collected after 36 h post-transfection and also on the day 4 of the transfection. Harvested lentivius-containing supernatant was then filtered through a 45 µm Polyvinylidene fluoride (PVDF) filter (Thermo Fischer Scientific). Two mL of Lenti-X™ Concentrator (Clontech; Mountain View, CA, USA) was added to 6 ml of this filtrate and incubated overnight. The reaction was then centrifuged at 1500× *g* for 45 min at 4 °C. Supernatant was removed carefully without touching the precipitate and resuspended in 2 mL of the PBS. The PBS containing the virus was stored at −80 °C for further use. Two hundred µL from this solution was used to perform RT-PCR to check for the presence of the WPRE region as a secondary confirmation for the virus production.

### 3.4. Virus Confirmation Using Reverse Transcription Polymerase Chain Reaction (RT-PCR)

After the viral production, RT-PCR was performed to confirm the presence of viral components, as the CRISPR plasmids did not have a fluorescent marker. The concentrated virus was lysed using Nucleus Lysis Solution (NLS Buffer), 0.5% ethane-1,2-diyldinitrilo) tetra acetic acid (EDTA) and water. DNA was extracted from these samples to run RT-PCR using the primers, which specifically amplify unique lentiviral portions, namely WPRE. The RT-PCR reactions consisted of 1 μL of DNA, 1 μL of the 20 μM forward primers 5′-GTCCTTTCCATGGCTGCTC-3′ and 5′-CCGAAGGGACGTAGCAGA-3′ reverse primer, 12.5 μL of RT-PCR SYBR green master mix (Biorad; Hercules, CA, USA), and 10.5 μL water. The thermal cycler was set at 95 °C for 10 min to activate the polymerase enzyme during the first step. The second step was consisted of a 15 s incubation period at 95 °C, followed by 1-min incubation at 60 °C, with this process being repeated through a total of 40 cycles.

### 3.5. Isolation of Mesenchymal Stem Cells (MSCs)

MSCs were isolated from the bone marrow of the femur of YAC128 mice (genotype confirmed using 94–96 primers as recommended by Jackson laboratories (Bar Harbor, ME, USA). Whole marrow was extracted via aspiration in a 25-G needle and re-suspended in 10 mL MSC media, which contained α Minimal Essential Medium (αMEM; Gibco^®^; Waltham, MA, USA), supplemented with 10% fetal bovine serum (heat inactivated), 10% horse serum (HS; Gibco^®^; Waltham, MA, USA), and 5 mg/mL streptomycin, and 5 UI/mL penicillin (Sigma). The cells were collected in a 15 mL Falcon tubes and were centrifuged at 300× *g* for 7 min at 4 °C. The MSCs were then counted and plated in a 25 cm^2^ flask (MidSci; St. Louis, MO, USA) containing 5 mL of MSC medium. After incubation for 48 h at 37 °C and 5% CO_2_, the media was replaced with fresh MSC media to remove non-adherent cells. MSCs were passaged with 0.25% Trypsin-EDTA (Gibco^®^; Waltham, MA, USA), when 80%–85% confluency was reached.

### 3.6. Lentiviral Transduction into the MSCs

The MSCs were titrated with 10-fold gradient concentration (1–1000 µL/well) of the purified virus, along with 6 μg/mL polybrene (Sigma), in order to determine safe level of virus at maximum efficiency. For this, BM MSCs (6.5 × 10^4^/mL) were suspended in 1 mL of stem cell media, and plated on pre-coated coated 6-well plates with 0.01% Poly-d-Lysine (Sigma). The required amount of the virus (1-, 10-, 100-, or 1000-µL/well) was added to 1 mL of the media, along with 6 µg/mL Polybrene, followed by a 2-min incubation at room temperature. This mixture was then added to each well. After 10 h post-virus application, the media was replaced with fresh stem cell media. We included a GFP-lenti virus, which was produced in parallel with the Lenti-CRISPR-gRNA1 and Lenti-CRISPR-gRNA2, as a control. It was observed that the wells containing 1000 µL of eGFP lentivirus, the cell death was about 80%, with the remaining cells showing signs of an unhealthy morphology, whereas, at 100 µL/well, there was 70% cell survival with little change in morphology. Cells with 10 µL virus and 1 µL virus resulted in no difference when compared with control BM-MSCs. After 3 days of post-transfection, 35%–40% BM- MSCs emitting fluorescence could be visualized in wells loaded with 100 µL of the eGFP. Similar effects were observed in the Lenti CRISPR-gRNA1 and Lenti-CRISPR-gRNA2 virus, correlating to the concentration of the virus. In order to study a wide range of the resulting CRISPR-Cas9-mediated indel-mutation at the targeted region, we plated cells in 10 cm^2^ petri dish, which was 5.8 times larger than the area of the well in a six-well plate. We followed the same steps as described above, until the infected cells reached 80%–90% confluency (3–5 days). Puromycin (Sigma) was applied to these cell cultures on day 5 (at a concentration of 5 µL/mL), while changing the media on alternate days until the fifth passage. The main reason behind the extended puromycin treatment was to ensure that pure lentivirus was infecting the cells, as the Lenti-CRISPR v2-gRNA plasmids do not have a florescent marker to determine the point-of-complete selection. After 2 passages, it was observed that 100% of the BM-MSCs treated with eGFP showed a fluorescent label under FITC filter (Figure S2).

### 3.7. mHTT Gene Sequencing

After puromycin selection, gRNA1 and gRNA2-treated cells were plated separately at 5 × 10^5^/mL in a 75 mL-flask to test the gene-editing. DNA from these cells was extracted using TRIzol^®^ reagent (Life technologies; Invitrogen, CA, USA), according to the manufactures protocol. To study the gene editing, we amplified the region around the DSB, with forward primer 5′-CCGCTCAGGTTCTGCTTTTA-3′ and reverse primer 5′-GAGTCCCTCAAGTCCTTCCA-3′ to amplify the DNA at gRNA1-target (Figure S3. A). To study editing at gRNA2-target site, forward 5′-CCTCCTCAGCTTCCTCAGC-3′, and reverse primer 5′-CCTCACTTGGGTCTTCCCTTGT-3′ were used (Figure S3. B). The amplified DNA was gel-purified and ligated with T-Easy vector before transformation into JM109 competent cells (Promega; Madison, WI, USA). Colonies were randomly picked and sequenced at Eton Bioscience (San Diego, CA, USA).

### 3.8. qRT-PCR

The media from puromycin-selected gRNA1 and gRNA2-treated cells was removed and were washed twice with PBS. YAC128 BM-MSCs (before gRNA treatment) were used as control in all the experiments. Two mL TRIzol^®^ reagent was added per to each flask, and incubated for five minutes, to completely lyse the cells. The lysates were stored at −80 °C until RNA isolation was performed. RNA was isolated from the lysate by following the manufacturer’s protocol. Briefly, samples were thawed and incubated at room temperature for five minutes. Cells were then homogenized by adding 0.2 mL of chloroform (Sigma) to each 1 mL of TRIzol^®^ reagent, and the samples were mixed for 15 s and incubated for 2–3 min at room temperature. Samples were then centrifuged at 12,000× *g* for 15 min at 4 °C, and the aqueous phase of the samples was removed and placed into new tubes, following centrifugation. Samples were then incubated in 500 µL of 100% isopropanol (Sigma) for 10 min, followed by centrifugation for an additional 10 min. The supernatant was removed from the tube and the remaining RNA pellet was washed with 1 mL of 75% ethanol (Sigma), vortexed for 10 s, and centrifuged at 7500× *g* for 5 min. Ethanol was discarded and the RNA pellet was allowed to air dry for 20–30 min before it was re-suspended in 30 µL of RNase-free water (Qiagen; Valencia, CA, USA).

Following RNA isolation, the iScript Advanced cDNA Synthesis Kit for qRT-PCR (Biorad) was used to transcribe complimentary DNA (cDNA) from the RNA. The cDNA was utilized in a series of RT-PCR reactions to quantify mRNA expression levels of m*HTT* gene in exon 2–4 using forward primer 5′-GAAAACATAGTGGCACAGTCTG-3′ and reverse primer: 5′-CTCGAGCTGTAACCTTGGAAG-3′. The qRT-PCR reactions were performed in replicates. Each reaction consisted of 1 µL diluted cDNA, 0.5 µL of the 20 µM reverse and forward primers, and 10 µL iQ™ SYBR^®^ Green Supermix (Biorad). Reactions were loaded into 92-well RT-PCR plates and centrifuged at 4000× *g* for 5 min at 4 °C to uniformly distribute each reaction within all of the wells. The RT-PCR reactions were then performed. First, the samples were placed in a thermal cycler and incubated at 95 °C for 10 min to activate the DNA-polymerase enzyme. Next, they underwent 40 cycles of a denaturing stage (95 °C for 15 s) and an annealing stage (60 °C for 1 min), during which time a camera was set to record the reaction. Relative mRNA expression was analyzed using the comparative threshold cycle (*C*_T_) method or the 2^−ΔΔ*C*^_T_ method, which presents the fold change in relative gene expression between a control and treatment sample. First, mRNA from each sample (i.e., control MSCs CRISPR-gRNA1 and CRISPR-gRNA2) was normalized to a house keeping gene, or internal control, to obtain Δ*C*_T_, and the fold change due to treatment was determined by ΔΔ*C*_T_ (2^−ΔΔ*C*^_T_) [44].

### 4.9. Detection of mHTT Level Using Western Blot

Gene-edited-MSCs were plated in a 60-mm^2^ Petri-dish at 1 × 10^5^ cells/mL. When the growth of MSCs reached 80% confluency, the media was discarded, and the cells were washed three times with sterile cold Tris buffer saline (TBS; 25 mM Tris, 150 mM NaCl, 2 mM KCl, at pH = 7.4) and scraped in the same buffer, followed by the cell suspension and centrifugation at 1000× *g* for 5 min at 4 °C. The MSCs that contained the transgene were plated to run as a control through the process. The protein of these cells was extracted using radio immunoprecipitation assay (RIPA) buffer with proteases and phosphatases inhibitors cocktail. The supernatant was aliquoted into small Eppendorf tubes and were stored at −80 °C until their use. The total protein was estimated using BCA kit, where bovine serum albumin (BSA; Pierce™, Thermo Fisher Scientific; Waltham, MA, USA) was used as a standard. The lysates were boiled with sample loading buffer (composition) and separated in 8% sodium dodecyl sulphate-polyacrylamide gel electrophoresis (SD-PAGE; Biorad), and then transferred onto poly-vinylidene fluoride (PVDF) membrane. The membrane was probed to detect human HTT, with anti-huntingtin mouse monoclonal antibody EM48 (MAB5374, EMD Millipore; Jaffrey, NH, USA) at a dilution of 1:500 in 5% milk, and the signal was detected using chemiluminescent kit (Pierce Biotec; St. Louis, MO, USA), following incubation with the appropriate secondary antibody. Then, the same blot was probed with rabbit monoclonal anti-β-tubulin antibody # 5346 (Cell Signaling Technology, Inc., Danvers, MA, USA) for the loading control. The optical density (OD) of each band was measured using ImageJ 1.50i software (https://imagej.nih.gov/ij/).

### 3.10. Statistical Analysis

All the data were expressed as mean ± SEM. One-way analysis of variance (ANOVA), followed, when appropriate, with Tukey’s Honestly Significant Difference (HSD) *posthoc* tests or with pairwise comparisons using Bonferoni corrections, were performed to provide statistical comparisons among the groups. The *p*-value ≤ 0.05 was considered to be statistically significant.

## 4. Conclusions

Results from this study demonstrate that CRISPR-Cas9 mediated mHTT editing upstream of the ORF, and that the exon1-intron1 region profoundly affects transcription of the *HTT* gene in vitro. However, when compared to editing the exon1-intron junction of the *HTT* gene, editing of the untranslated region produced greater efficacy in silencing the gene. Our study also emphasizes that NHEJ can be used to produce a large number of nucleotide deletions at the targeted region. Our future studies will be focused on achieving specificity towards editing the mutant allele, while sparing the normal allele. We believe that CRISPR-Cas9 strategy has the advantage of providing permanent gene-silencing that can be achieved at higher efficiency, and done more economically than the other gene editing strategies, providing a means whereby allografts of “gene-silenced” cells might be safely and effectively used for transplantation into HD patients.

## Figures and Tables

**Table 1:** Oligo sequences for cloning

Target	Primer Sequence
Untranslated region(gRNA1)	5′-CACCGGCCTCCGGGGACTGCCGTGC-3′5′-CCGGAGGCCCCTGACGGCACGCAAA-3′
Exon1-intron(gRNA2)	5′-CACCGGGTTCGTGTCGCCGGCCCGC-3′5′-CCCAAGCACAGCGGCCGGGCGCAAA-3′

## Supplementary Materials

The following are available online at www.mdpi.com/1422-0067/18/4/754/s1.
