# Supplementary material for: CRISPR-Cas9 Mediated Gene-Silencing of the Mutant Huntingtin Gene in an In Vitro Model of Huntington’s Disease
Source: Int J Mol Sci. 2017 Apr 2;18(4):754. doi: 10.3390/ijms18040754 (PMC5412339; doi:10.3390/ijms18040754)
Supplement: Supplementary file 1 [file ijms-18-00754-s001.pdf]

Supplementary material

Table S1: Potential off-target effects with gRNA1.

sequence	mismatches	UCSC gene	locus	Location
GCAAGGCTGTCCCCGGAGGCGGG	2MMs [4:8]		chr19:+2215587	Intron
GCACGCCAGGCCCCGGAGGCGGG	2MMs [6:10]	NM_006039	chr17:-60758088	Exon
GCCAGGCAGGCCCCGGAGGCAGG	3MMs [3:4:10]		chr22:-47431106	Intron
ACACGGCAGTCCCAGGAGGCTGG	2MMs [1:14]		chr1:-1557524	Intron
TCCCGTCAGTCCCCGGAGGCCAG	3MMs [1:3:6]	NM_133475	chr19:+4212461	Exon
GCGCGGCGGTGCCCGGAGGCTGG	3MMs [3:8:11]		chr5:-176245036	Intron
GGACGGCACTCCCCGGAGGTAAG	3MMs [2:9:20]	NM_015073	chr19:+38621393	Exon
GCCTGGCAGTCCCCTGAGGCTGG	3MMs [3:4:15]		chr22:-39924296	Intron
GGACGGAAGTCCCCAGAGGCTAG	3MMs [2:7:15]		chr2:-233227947	Intron
GCACGTGAGTCTCCGGAGGCCGG	3MMs [6:7:12]		chr9:+140209299	Intron
ACCCGGCAGTCCCAGGAGGCCAG	3MMs [1:3:14]		chr1:-6324894	Intron
GCACGGCCCTCCCCAGAGGCCAG	3MMs [8:9:15]		chr13:-114444313	Intron
GCACGCCAGGCCCCCGAGGCTGG	3MMs [6:10:15]		chr5:-62015504	Intron
GCACGGCAGCCTCCGGAGCCAGG	3MMs [10:12:19]	NM_023068	chr20:+3673382	Exon
GCCCGGCAGGCCCAGGAGGCGGG	3MMs [3:10:14]	NM_170600	chr9:-130536477	Exon
GCAGGGCAGTCCCCCGAGGGCAG	3MMs [4:15:20]		chr11:+2792144	Intron

Table S2: Potential off-target effects with gRNA2.

sequence	mismatches	UCSC gene	locus	Location
GAGGGGCGGGGACACGAACGAGG	4MMs [2:6:10:20]	NM_031461	chr8:-75896816	Exon
CAGGCCCGGCGACACCAACCTGG	4MMs [1:2:5:16]		chr6:+34051008	Intron
GAGGCACGGCGACAAGAACCCAG	4MMs [2:5:6:15]		chr22:-40644222	Intron
CGGGGCCGGCCACAAGAACCCAG	4MMs [1:2:11:15]		chr8:-61948047	Intron
GAGGGCAGGCCACACGCACCGGG	4MMs [2:7:11:17]	NM_005560	chr20:+60888362	Exon
GCGAGGCGGCCACACGCACCGAG	4MMs [4:6:11:17]	NM_004530	chr16:+55513325	Exon
GCAGGCCGGTGGCAGGAACCAGG	4MMs [3:10:12:15]	NM_198456	chrX:+54208976	Exon
GCGGGCCCTCGCCACGCACCCGG	4MMs [8:9:12:17]	NM_001520	chr16:-27561041	Exon
CAGGGCCGGCGCCACGACCCGGG	4MMs [1:2:12:18]		chr1:+159894869	Intron
CCGGGCCGGCGGGACGAACGCAG	4MMs [1:12:13:20]	NM_145239	chr16:-29827222	Exon
GCGGGGCAGGGACCCGAACCGGG	4MMs [6:8:10:14]		chr1:+15481403	Intron
GCAGGCCGGAGACACGTCCCCGG	4MMs [3:10:17:18]		chr1:-32403475	Intron
GCGGGCCAGCACCAGGAACCAGG	4MMs [8:11:12:15]	NR_027138	chr11:+3239526	Exon
GCGGGCGGGGGACAGGAAGCGGG	4MMs [7:10:15:19]		chr7:+1708958	Intron
GCAGGCCAGCGACTCGAAACCGG	4MMs [3:8:14:19]	NM_000465	chr2:+215674364	Exon
GCTGGCCGGCGACAGGGACAAAG	4MMs [3:15:17:20]		chr5:+176293908	Intron
GCGGGGCGGGGGCACAAACCAAG	4MMs [6:10:12:16]		chr13:+28577006	Intron
GCTGCCCGGCGACACAAGCCCAG	4MMs [3:5:16:18]		chr8:-33267921	Intron
GCGGGCCGGGTCCTCGAACCTGG	4MMs [10:11:12:14]		chr15:-51386361	Intron
GCGGGCCTGCGGCCCGGACCCGG	4MMs [8:12:14:17]	NM_015641	chr7:-115850567	Exon
GGGGGCCGGCGACACAAAGGCGG	4MMs [2:16:19:20]		chr17:+60555802	Intron
GCGGGCTGGCGGCACTCACCCAG	4MMs [7:12:16:17]		chr8:+7923764	Intron
GCGGGCGGGCGCCGCGAACATGG	4MMs [7:12:14:20]		chr7:-30028806	Intron

sequence	mismatches	UCSC gene	locus	Location
GCGGCCCGGCGACAGGGTCCGGG	4MMs [5:15:17:18]		chr15:-75494939	Intron
GCGGGCGGGCCACAAGAGCCGGG	4MMs [7:11:15:18]		chr5:+142783709	Intron
GCGGGCCGCCGTCACCATCCAGG	4MMs [9:12:16:18]	NM_004145	chr19:+17303681	Exon
GCGGGCCGGCGCCGCGGAGCGAG	4MMs [12:14:17:19]	NR_034172	chr17:+44271185	Exon
GCGGGCCGCCGACCGGAAGCGGG	4MMs [9:14:15:19]	NR_039924	chrX:+7065829	Exon
GCGGGGCGGCGACGCGGGCCCGG	4MMs [6:14:17:18]	NM_030786	chr1:+33168173	Exon

Supplemental figures

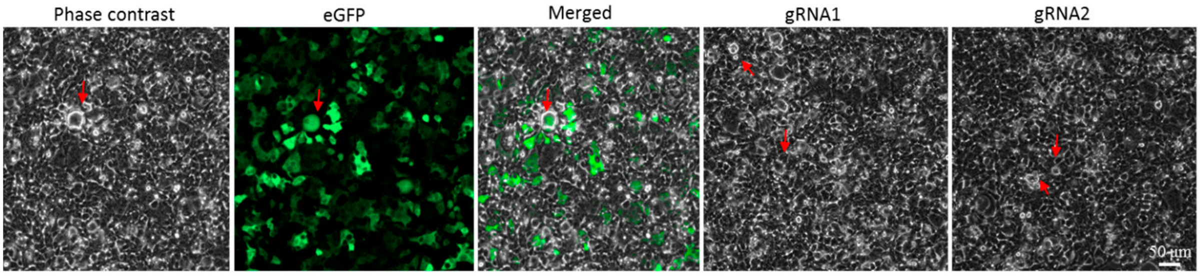

S1

Figure S1: **Morphological changes in 293FT cells after 24 h post-transfection.** The 293FT cells were grown in DMEM and transfected with PAX2, VSVG with either eGFP or gRNA1 or gRNA2 using lipofectamine-3000. Note that the 293FT cells showing 'balloon-like' morphology (highlighted with red arrow) 24 h post-transfection indicate successful production of lenti-virus. Scale bar is 50 µm and is applicable to all the images.

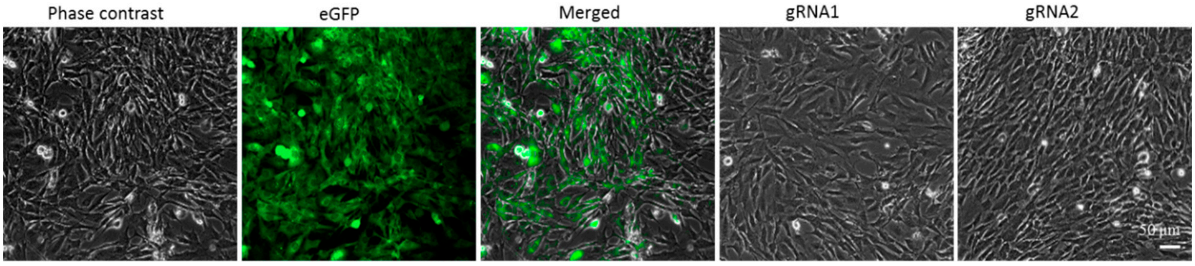

S2

Figure S2: Puromycin selected BM-MSCs. BM-MSCs were cultured in MEM-α and infected with lenti-eGFP or lenti-CRISPR gRNA1 or lenti-CRISPR-gRNA2. Resistance and the survival of infected BM-MSC cultures to puromycin exposure after 15 days post-infection were observed. Scale bar is 50 µm and is applicable to all images.

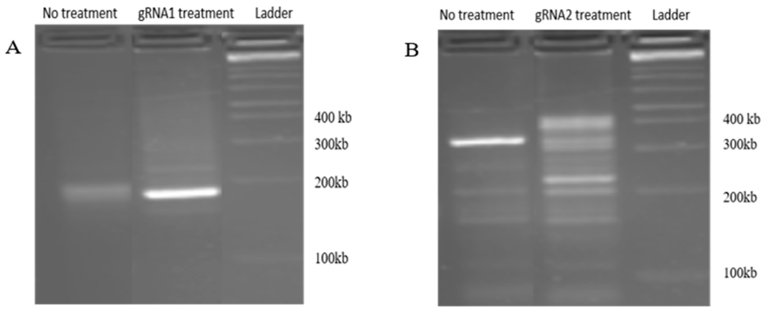

Figure S3: Amplification of the region around the double strand break (DSB). This figure shows the amplified region around the DSB, from the YAC128 BM-MSCs before and after treatment with lenti-CRISPR-gRNA1 (A) and lenti-CRISPR-gRNA2 (B). The product size expected for A is 179 kb, and B is 339 kb.
